# Development of innovative high-pressure processed olive pâtés containing pasteurized olive pomace paste: a study focusing on chemical and sensory properties

**DOI:** 10.3389/fnut.2026.1794079

**Published:** 2026-03-20

**Authors:** Diana Melo Ferreira, Maria Manuela Sousa, Susana Machado, Carla Barbosa, Carlos Pinto, Jorge A. Saraiva, Maria Beatriz P. P. Oliveira, Rita C. Alves

**Affiliations:** 1 REQUIMTE/LAQV, Faculty of Pharmacy, University of Porto, Porto, Portugal; 2 LAQV-REQUIMTE, Department of Chemistry, University of Aveiro, Campus Universitário de Santiago, Aveiro, Portugal

**Keywords:** by-product valorization, functional food, nutritional enhancement, plant-based food, sustainable food innovation

## Abstract

**Introduction:**

Pasteurized olive pomace paste (PastOPP) is a sustainable ingredient characterized by low fat content and high levels of dietary fiber and hydroxytyrosol. This study explores the potential of incorporating PastOPP into olive pâté formulations to enhance their nutritional profile.

**Methods:**

Laboratory-produced formulations containing different percentages of PastOPP (0, 20, and 25%: P0, P20, and P25, respectively) were compared to a commercial olive pâté (CP) regarding macronutrients, vitamin E (HPLC-DAD-FLD), fatty acids (GC-FID), total phenolics, and hydroxytyrosol (HPLC-DAD-FLD) contents, as well as antioxidant activity. High-pressure processing (HPP) was applied to lab-produced formulations, yielding P0*, P20*, and P25*. Sensory evaluation of the final products included a check-all-that-apply test.

**Results:**

HPP preserved all the tested parameters without significant changes (*p* > 0.05). CP showed lower moisture, protein, carbohydrate, salt, polyunsaturated fatty acids (PUFA), total phenolics, and antioxidant activity levels than the lab-prepared pâtés, while exhibiting higher fat, monounsaturated fatty acids (MUFA), and MUFA/PUFA ratios (*p* < 0.05). Hydroxytyrosol was undetectable in CP but present in significant amounts in P20* and P25*, which also featured higher fiber, vitamin E, and antioxidant activity levels. Sensory tests revealed that P20* and P25* were preferred over CP, with higher acceptability and purchase intention scores, but P20* was preferred over P25*.

**Conclusion:**

P20* and P25* represent innovative and sustainable alternatives to traditional olive pâtés, offering enhanced nutritional benefits and alignment with consumer demand for plant-based, functional, and eco-friendly food options. By valorizing an agricultural by-product, these formulations also contribute to circular economy practices.

## 1 Introduction

The global food supply chain faces major challenges, including rapid population growth, climate change, and unexpected crises (1–3). With the global population projected to reach 10 billion by 2050, food production will need to increase by 70%, placing immense pressure on the agricultural sector (1). Climate change exacerbates this issue through rising temperatures, extreme weather events, and soil degradation, further threatening food security (2). The COVID-19 pandemic also exposed vulnerabilities in food systems, highlighting the need for innovative and sustainable solutions (3).

In this context, innovative strategies such as the valorization of agri-food by-products have gained increasing attention as they provide opportunities to address these global challenges. Moreover, these approaches align with circular economy principles by reducing waste, promoting sustainability, and enhancing food security and economic resilience (4).

Within the food chain, the olive oil industry holds deep economic and cultural significance, particularly in Mediterranean countries like Portugal, where olive oil is a dietary staple. As olive oil production grows, so does the generation of by-products, such as olive pomace (OP), a semi-solid residue comprising olive skin, pulp, and stone. OP is rich in valuable compounds, including phenolic compounds, which have potential applications in the food industry (5–9).

OP is one example of a by-product that can be effectively exploited within such innovative strategies. It has already been incorporated into food products, with successful applications reported in pasta and bakery formulations (9–11). Additionally, OP contains a natural microbiota, which has been investigated through spontaneous fermentation processes, showing promising results (7). Due to its inherent microbial load, in a previous study, Sousa et al. (6) investigated different pasteurization times and temperatures to ensure microbial safety, identifying optimal conditions at 88 °C for 15 s. These conditions effectively reduced the microbial load while minimally affecting the bioactive profile. As a result, a pasteurized olive pomace paste (PastOPP) with functional and nutritional potential was obtained (6).

PastOPP is characterized by high moisture and considerable dietary fiber, along with residual lipids that provide vitamin E (particularly *α*-tocopherol) and beneficial fatty acids, including oleic acid, associated with cardiovascular health and food stability, and linoleic acid, which contributes to reducing total and LDL cholesterol levels. Additionally, PastOPP is rich in phenolics, mainly hydroxytyrosol, and flavonoids, conferring strong antioxidant properties. Thus, PastOPP could be a promising ingredient with high potential for industrial application in functional foods (6).

Indeed, the development of new food products is a critical task for the food industry, driven by consumer demand and technological advancements. The growing trend toward health-conscious and sustainable living has spurred interest in functional foods - products that offer health benefits beyond basic nutrition (12, 13). Similarly, the rise of plant-based diets, motivated by health, ethical, and environmental concerns, has created demand for innovative, sustainable, and convenient food options, despite challenges such as ingredient availability and sensory appeal (14). In this context, sensory analysis plays a crucial role in food product development, providing insights into consumer preferences and guiding product refinement. Techniques such as the check-all-that-apply (CATA) test and hedonic scales are essential for evaluating sensory attributes and consumer acceptability (15–18).

Considering these aspects, the previously described properties of PastOPP make it particularly suitable for incorporation into food products. Its inclusion can enhance dietary fiber content, antioxidant capacity, and the healthy lipid profile of foods, while meeting consumer demand for functional, plant-based, and sustainable products (6). In this context, olive pâté, a traditional Mediterranean spread made from olives, herbs, and spices, offers a promising opportunity to combine tradition with innovation by incorporating by-products (19). Indeed, previous studies have explored the incorporation of olive mill wastewater (another by-product of the olive oil industry) in olive pâté (20–23).

Taking all these considerations into account, the present study aimed to valorize PastOPP by developing a sustainable, functional, innovative, and convenient food product. Since PastOPP has not yet been incorporated into a food product, olive pâtés enriched with this ingredient were formulated. The objectives of the study included a comprehensive chemical characterization of PastOPP and the developed pâtés to understand the impact of PastOPP incorporation on their nutritional value, bioactive compounds content, and antioxidant profile. Furthermore, High-Pressure Processing (HPP) is a non-thermal food preservation technology that applies very high hydrostatic pressure (typically ranging from 100 to 600 MPa) to inactivate microorganisms and certain enzymes, without the use of heat. This approach helps preserve the nutritional and sensory qualities of foods, including vitamins, color, and flavor, which are often degraded by conventional thermal treatments. HPP offers several advantages, such as extended shelf life, enhanced food safety, and minimal impact on taste and texture. However, limitations including high equipment costs and restricted applicability to specific food matrices must also be considered. Currently, HPP is widely applied in products such as fruit juices, ready-to-eat meals, seafood, and meat products, demonstrating its increasing relevance in modern food processing (24). In the present study, HPP was applied to laboratory-produced olive pâtés containing PastOPP to investigate its effects on chemical composition, bioactive compound retention, antioxidant activity, and sensory attributes. This combined approach constitutes a key novelty of the work, as it explores the combined use of a sustainable, functional ingredient with advanced preservation technology to develop an innovative, high-quality, and health-promoting food product.

## 2 Materials and methods

### 2.1 Chemicals and reagents

The chemicals and reagents used were of analytical grade and sourced as follows: acetic acid (CH₃COOH), methanol (HPLC-grade), Kjeldahl tablets, sulfuric acid (H₂SO₄), sodium hydroxide (NaOH), anhydrous sodium sulphate (Na₂SO₄), sodium dihydrogen phosphate (NaH₂PO₄), di-sodium hydrogen phosphate anhydrous (Na₂HPO₄), silver nitrate (AgNO₃), potassium dichromate (K₂Cr₂O₇), *n*-hexane (HPLC grade), sodium chloride (NaCl), and sodium carbonate (Na₂CO₃) from Merck (Darmstadt, Germany). Boric acid 4% (H₃BO₃) and potassium hydroxide (KOH) were from Panreac (Barcelona, Spain). Sand was obtained from VWR Chemicals (Alfragide, Portugal), and petroleum ether and chloridric acid (HCl) from Carlo Erba Reagents (Val de Reuil, France). The fibre enzymes kit, celite, BHT (butylated hydroxytoluene), dioxane, Folin–Ciocalteu reagent, gallic acid, 2,4,6-tripyridyltriazine (TPTZ), ferric chloride (FeCl₃), heptahydrate ferrous sulphate, sodium acetate, 2,2-diphenyl-1-picrylhydrazyl radical (DPPH^●^), Trolox, and hydroxytyrosol standard were from Sigma-Aldrich (St. Louis, USA). Acetone was from Fischer Chemical (UK), ethanol 96% from AGA (Prior Velho, Portugal), and absolute ethanol from both Merck (Darmstadt, Germany) and Sigma-Aldrich (St. Louis, USA). Tocopherols/tocotrienols standards were acquired from Calbiochem (La Jolla, CA, USA), and tocol was from Matreya Inc. (Pennsylvania, USA). Dichloromethane (CH₂Cl₂) was supplied by Honeywell Riedel-de HaënTM (Germany). Boron trifluoride-methanol solution (14% BF₃) and fatty acids standard (FAME37 Mix) were purchased from Sigma-Aldrich (St. Louis, USA). Ultra-pure water was obtained using a Milli-Q water purification system (Millipore, Bedford, MA, USA).

### 2.2 Samples

Olive pomace (OP) was collected in a two-phase olive mill in Alfândega da Fé, Portugal. Stone fragments were manually removed using a stainless-steel sieve, resulting in the obtention of olive pomace paste (OPP). OPP was then pasteurized using a Thermomix TM 31® (Vorwerk, Wuppertal, Germany) at 88 °C for 15 s to ensure microbiological safety, producing pasteurized olive pomace paste (PastOPP) as optimized in a previous study (6).

Three olive pâtés were prepared in the laboratory, using a Thermomix TM 31® (Vorwerk, Wuppertal, Germany). The control (P0) consisted of 75% black olives, 10% extra-virgin olive oil (EVOO), 10% vinegar, 3% aromatic herbs (oregano, chives, and basil), 1% garlic powder, and 1% salt (25). Two additional pâtés were produced by partially replacing olives with PastOPP: P20 with 20% PastOPP and 55% olives, and P25 with 25% PastOPP and 50% olives. The preparation followed the technological sequence: draining the olives (5 min), triturating them (10 min, velocity 10), adding PastOPP, vinegar, and EVOO, and mixing again (10 min, velocity 10). Aromatic herbs, garlic powder, and salt were then incorporated, followed by a final mixing step (10 min, velocity 10). The pâtés (P0, P20, and P25) were packed in vacuum in polyamide/polyethylene (PA/PE) bags for processing by high-pressure processing (HPP) at 550 MPa and 10 °C for 3 min, using a pilot-scale high hydrostatic pressure equipment with a 55 L vessel (Hiperbaric 55, Burgos, Spain), resulting in the following HPP-processed pâtés: P0*, P20*, and P25*.

In addition, a commercial olive pâté (CP) available in the Portuguese market, containing black olives (83%), olive oil, honey and aromatic herbs, was acquired for comparison purposes.

### 2.3 Moisture and macronutrients contents

A KERN DBS infrared balance (Balingen, Germany) was used to determine the moisture content. The determination of the contents of total ash (AOAC 920.153), total fat (AOAC 991.36), total protein (AOAC 928.8, using the nitrogen conversion factor of 6.25 (26)), and total dietary fibre (AOAC 985.29) was accomplished according to AOAC methods (27). The remaining carbohydrates were determined by difference (26).

### 2.4 Salt content

The salt content was determined using the following equation: Salt = Sodium × 2.5 (28). To obtain the sodium content, the chloride ion (Cl^−^) content was determined according to Lutz (1985) (29). First, samples were incinerated according to the total ash method (AOAC 920.153) (27). To dissolve the white ashes, 3 portions of 30 mL of hot deionized water were added to the crucibles. This solution was then transferred to a volumetric flask. After cooling, the volumetric flask was filled to the calibration mark. Then, the volumetric flask solution was filtrated (Whatman No. 4 filter paper) to an Erlenmeyer flask. A 10 mL aliquot was transferred into a new Erlenmeyer flask. Then, 2 drops of potassium chromate (10%) were added. Finally, the solution was titrated with silver nitrate (0.1 M) until a brick-red precipitate appeared.

### 2.5 Lipid fraction extraction

The lipid fraction was extracted in triplicate as described by Sousa et al. (6). Briefly, an appropriate amount of each sample containing 20 mg of fat was mixed with 50 μL of tocol (0.1 mg/mL) and 1 mL of absolute ethanol, followed by agitation for 30 min. Subsequently, 2 mL of *n*-hexane (HPLC grade) and 1 mL of 1% NaCl solution (m/v) were added sequentially, with agitation for 30 min and centrifugation steps to extract the fat-soluble components. A second extraction was performed on the residue using 2 mL of n-hexane, after which the organic phases were combined. Residual water was removed using anhydrous sodium sulfate (Na₂SO₄), and the *n*-hexane extract was used for analysis.

#### 2.5.1 Vitamin E profile

Vitamin E profile of the *n*-hexane extracts was determined using an HPLC system (Jasco, Tokyo, Japan), according to the conditions described by Sousa et al. (6). The system was equipped with a MD-2015 multiwavelength diode array detector coupled to a FP-2020 fluorescence detector, programmed at λ_excitation_ = 290 nm, and λ_emission_ = 330 nm (both from Jasco, Tokyo, Japan). A normal phase Supelcosil™ LC-Si HPLC Column (3 μm particle size, L x I. D. 15 cm x 4.6 mm) was used to separate the compounds. In addition, a standard stock solution containing individual compounds was prepared to obtain calibration curves for each vitamer (*α*-tocopherol: y = 0.1757 x − 0.0470; α-tocotrienol: y = 0.2106 x − 0.0546; *β*-tocopherol: y = 0.2340 x − 0.0406; *γ*-tocopherol: y = 0.1622 x − 0.0437; β-tocotrienol: y = 0.1802 x − 0.0411; γ-tocotrienol: y = 0.1593 x − 0.0505; *δ*-tocopherol: y = 0.1760 x − 0.0324; and δ-tocotrienol: y = 0.1684 x − 0.0005). The injection volume was 20 μL, eluted with 6% dioxane in *n*-hexane (HPLC grade), at a flow rate of 0.7 mL/min. The different vitamin E vitamers (*α*, *β*, *γ*, δ-tocopherols and tocotrienols) were identified based on the UV spectra and on the comparison of their retention times with those of standards. Moreover, their fluorescence signals were used to quantification.

#### 2.5.2 Fatty acids profile

Fatty acids were derivatized into fatty acid methyl esters (FAME) following the ISO 12966-2:2017 method (30). A Shimadzu GC-2010 Plus gas chromatograph coupled with a flame ionization detector (GC-FID, Tokyo, Japan) was used to obtain the fatty acids profile, according to the conditions described by Sousa et al. (6). FAME identification was carried out by comparison of their retention times with standards (Supelco 37 Component FAME Mix), being the data examined based on relative peak areas.

### 2.6 Extraction of bioactive compounds

Extracts were obtained in triplicate. Briefly, 100 mg of each sample were mixed with 40 mL of an ethanol and water mixture (70/30, v/v), under constant stirring, for 60 min, followed by centrifugation (4,696 × g, 5 min) and the supernatant was used for analysis.

#### 2.6.1 Total phenolics content (TPC) and antioxidant activity assays

TPC was determined as described by Sousa et al. (6). A gallic acid calibration curve was also prepared (y = 0.009 x − 0.004; R^2^ = 0.999). Results are presented in gallic acid equivalents (GAE).

Ferric reducing antioxidant power (FRAP) assay was determined according to Sousa et al. (6). A ferrous sulphate (FeSO_4_) calibration curve was also prepared (y = 0.002 x − 0.016; R^2^ = 0.999) and results presented in ferrous sulphate equivalents (FSE).

2,2-Diphenyl-1-picrylhydrazyl radical scavenging activity (DPPH^●^-SA) assay was determined according to Sousa et al. (6). A Trolox calibration curve was prepared (y = −0.006 x + 0.5278; R^2^ = 0.999) and results presented in Trolox equivalents (TE).

#### 2.6.2 Hydroxytyrosol content

Hydroxytyrosol content was determined by injecting 1 mL of extract (see 2.6) into an HPLC system (Jasco, Tokyo, Japan) composed of a multiwavelength diode-array detector (MD-2018 Plus) coupled to a fluorescence detector (FP-2020 Plus). A Zorbax-SB-C18 chromatographic column (250 × 4.6 mm, 5 μm, Agilent Technologies, Netherlands) at 20 °C was used. A gradient elution program was employed, using acetic acid (A, 1%) and methanol (B, 100%): 0 min, 5% B; 30 min, 25% B; 50 min, 75% B; 55 min, 100% B; 63 min, 5% B. The flow rate was 1 mL/min. The injection volume was 20 μL. Hydroxytyrosol content was evaluated by fluorescence and monitored at λ_excitation_ = 280 nm and λ_emission_ = 330 nm.

### 2.7 Sensory evaluation

In addition to the HPP-processed olive pâtés, a commercial olive pâté (CP) available in a Portuguese supermarket was included in the sensory evaluation for comparison purposes. A check-all-that-apply (CATA) analysis (17) with 34 attributes grouped by sensory modality and listed alphabetically was used for the sensory evaluation of the pâtés. Each one of the 100 consumers received the pâtés coded with three random numbers (613 - P0*, 278 - P20*, 157 - P25*, and 831 - CP) at room temperature, a glass of water, soda crackers, a butter knife, a napkin, a pen, and a questionary. Consumers were instructed to analyse each sample once, cleansing the palate with water between samples. The participants’ identities remained anonymous, and the involvement in the study was voluntary. Participants were also informed that their involvement implied consent to use their responses for research purposes.

First, participants provided their demographic information (age, gender, and occupational status) in the questionnaire, followed by sensory attributes regarding general appearance (dull, shiny, light, dark, grainy, and oily), odor (aromatic, vinegary, oil, olive, spicy, and rancid), and texture when spread on a cracker (spreadable, creamy, grainy, liquid, smooth, and sticky). After tasting, tasters evaluated the flavor (acidic, astringent, bitter, vinegary, oil, olive, spices, very bitter, spicy, rancid, and salty) and texture in the mouth (consistent, creamy, grainy, liquid, and sticky). Then, tasters evaluated the overall acceptability, assessing their overall appreciation on a 9-point hedonic scale: 1 - “Dislike extremely” to 9 - “Like extremely” (15). Afterwards, tasters evaluated the purchase intention, using a 5-point hedonic scale: 1 - “Definitely will not buy” to 5 - “Definitely will buy” (31). Finally, they were requested to select all the attributes they considered necessary for an ideal olive pâté, concerning general appearance, odor, texture after spreading, flavor, and texture in the mouth (16).

The sensory evaluation involved voluntary participation of anonymous consumers, and no personally identifiable information was collected. All participants were informed about the purpose of the study and provided free informed consent before participation. The Ethics Committee of the Faculty of Pharmacy of the University of Porto (CEFFUP) confirmed that the main ethical requirements had been adequately safeguarded.

### 2.8 Statistical analysis

Significant differences between the results from the chemical analysis of the samples were assessed using a one-way ANOVA test, followed by a Tukey HSD post-hoc test, with a significance level of 0.05. The IBM SPSS v. 28 (IBM Corp., USA) was used for data analysis.

The software STATISTICA v.14 (Stat Soft Inc., Tulsa, USA) was used to perform the principal component analysis (PCA) and the correspondence analysis (CA) to investigate the acceptability and purchase intention differences between pâtés. Data were assessed by Kruskal-Wallis H-test (95% confidence level), followed by multiple comparison Wilcoxon/Mann-Whitney post-hoc tests (32), as data showed a non-parametric behavior. Boxplots were also obtained using this software.

In the CATA test, significant differences between pâtés for each attribute were assessed by a Cochran’s Q test followed by multiple pairwise comparisons using the Critical difference (Sheskin) procedure, with a level of significance of 0.05, and using XLSTAT 2022 (Addinsoft, Paris, France) (33).

## 3 Results and discussion

### 3.1 Nutritional composition

Olive pomace (OP) is a semi-solid by-product recognized as a rich source of valuable compounds with potential applications in the food industry (5–9). However, its high native microbial load limits its direct incorporation into food products. To address this limitation, a previous study evaluated different pasteurization time–temperature combinations and identified optimal conditions at 88 °C for 15 s, which effectively reduced microbial load while preserving the bioactive profile (6).

In the present study, these conditions were applied to obtain a pasteurized olive pomace paste (PastOPP) suitable for food use. As a first step, the chemical and nutritional composition of the PastOPP used for incorporation into olive pâté formulations was evaluated. The nutritional composition of PastOPP is presented in Table 1. As can be observed, PastOPP presented a high moisture content (65% fw) and was rich in carbohydrates (15% fw). Overall, the high fiber (12% fw) and low fat (2.5% fw, 77% of which is oleic acid) contents are notable features of this ingredient. This is further complemented by the presence of *α*-tocopherol (1.2 mg/100 g fw) and hydroxytyrosol (0.2 g/100 g). In addition, significant antioxidant activities were obtained by FRAP (1.6 g FSE/100 g) and DPPH^●^-SA (0.6 g TE/100 g) assays, partially justified by the high TPC (1.5 g GAE/100 g). However, this richness in phenolics may also contribute to a distinct bitterness and astringency, representing a potential sensory challenge for product development (9). Indeed, several phenolic compounds identified in OP (including oleuropein-aglycone, hydroxyoleuropein, oleoside 11-methyl ester, ligstroside-aglycone, hydroxytyrosol-glucoside, caffeic acid, caffeoylglucoside, caffeoyl-6′-secologanoside, quercetin glucoside, apigenin-7-O-rutinoside, and 3,4-dihydroxyphenylglycol) have been linked to bitterness and astringency due to their strong interactions with salivary proteins (7, 8, 34). Although these compounds are nutritionally advantageous due to their antioxidant properties, their presence may negatively affect consumer acceptability. To mitigate these sensory challenges, aromatic herbs, garlic powder, and salt were incorporated into the olive pâté formulations developed in the present study. In addition to enhancing flavor complexity and potentially masking the astringent and bitter notes of PastOPP, herbs and spices are also recognized for their antioxidant and anti-inflammatory properties, further contributing to the functional value of the final product (35). The developed olive pâtés were evaluated using the same parameters applied to PastOPP. The full list of ingredients used for the formulation of the pâtés is detailed in the Section *2.2. Samples*.

**Table 1:** Nutritional composition of pasteurized olive pomace paste (PastOPP).

PastOPP	Dry weight	Fresh weight
Moisture (g/100 g)	–	65.3 ± 0.1
Protein (g/100 g)	6.7 ± 0.2	2.3 ± 0.1
Fat (g/100 g)	7.2 ± 0.2	2.5 ± 0.1
Ash (g/100 g)	7.7 ± 0.3	2.7 ± 0.1
Total fiber (g/100 g)	35.0 ± 0.6	12.2 ± 0.2
Remaining carbohydrates (g/100 g)	43.4 ± 0.4	15.1 ± 0.2
Vitamin E (mg/100 g)		
α-Tocopherol	3.39 ± 0.04	1.18 ± 0.01
*β*-Tocopherol	0.137 ± 0.001	0.048 ± 0.001
*γ* -Tocopherol	0.201 ± 0.001	0.070 ± 0.001
∑Tocopherols	3.73 ± 0.04	1.30 ± 0.01
TPC (g GAE/100 g)	4.3 ± 0.2	1.5 ± 0.1
Hydroxytyrosol (g/100 g)	0.49 ± 0.03	0.17 ± 0.01
FRAP (g FSE/100 g)	4.5 ± 0.3	1.6 ± 0.1
DPPH^•^-SA (g TE/100 g)	1.7 ± 0.3	0.6 ± 0.1
Fatty acids	Relative %	
Palmitic acid (C16:0)	10.29 ± 0.04	
Palmitoleic acid (C16:1)	0.44 ± 0.01	
Heptadecanoic acid (C17:0)	0.11 ± 0.01	
Stearic acid (C18:0)	2.6 ± 0.1	
Oleic acid (C18:1n9c)	77.3 ± 0.2	
Linoleic acid (C18:2n6c)	7.9 ± 0.2	
Arachidic acid (C20:0)	0.29 ± 0.02	
α-Linolenic acid (C18:3n3)	0.64 ± 0.03	
cis-11-Eicosenoic acid (C20:1n9)	0.19 ± 0.01	
Behenic acid (C22:0)	0.19 ± 0.01	
Lignoceric acid (C24:0)	0.10 ± 0.01	
∑SFA	13.6 ± 0.1	
∑MUFA	77.9 ± 0.2	
∑PUFA	8.5 ± 0.2	
MUFA/PUFA	9.2 ± 0.2	

The results are presented as mean ± standard deviation. TPC, Total phenolic content; GAE, Gallic acid equivalents; FRAP, Ferric reducing antioxidant power; FSE, Ferrous sulphate equivalents; DPPH^•^-SA, 2,2-Diphenyl-1-picrylhydrazyl radical scavenging activity; TE, Trolox equivalents; SFA, Saturated; MUFA, Monounsaturated; PUFA, Polyunsaturated fatty acids.

HPP offers several advantages: it can effectively extend the shelf life of the product without the addition of synthetic preservatives and preserve heat-sensitive bioactive compounds (such as vitamin E, hydroxytyrosol, and flavonoids), while maintaining the natural color, flavor, and texture of the product. Compared to conventional thermal treatments, HPP is expected to better preserve the antioxidant capacity and fatty acid profile of the pâtés, ensuring that the functional benefits of PastOPP are fully preserved. However, HPP has the limitation of a high initial investment cost (24). In this study, HPP was applied to laboratory-produced olive pâtés enriched with PastOPP as a processing strategy, aiming to assess its potential impact on product composition. Table 2 presents the nutritional composition of all pâtés, both before and after this HPP treatment. CP exhibited significantly lower moisture (54% fw), protein (1.1 g/100 g fw), carbohydrates (5.0 g/100 g fw), and salt (1.2 g/100 g fw) contents comparable to those of the lab-produced pâtés (P0, P0*, P20, P20*, P25, and P25*). However, it registered a significantly higher fat content (30.2 g/100 g fw).

**Table 2:** Nutritional composition of olive pâtés: CP (commercial), P0 (control), P0* (control processed by HPP), P20 (with 20% PastOPP), P20* (with 20% PastOPP processed by HPP), P25 (25% PastOPP), and P25* (25% PastOPP processed by HPP).

Sample	CP	P0	P0*	P20	P20*	P25	P25*
Fresh weight							
Moisture (g/100 g)	53.9 ± 0.5^b^	59.0 ± 0.8^a^	59.0 ± 0.8^a^	58.9 ± 0.9^a^	59.6 ± 0.5^a^	59.2 ± 0.5^a^	59.1 ± 0.4^a^
Protein (g/100 g)	1.11 ± 0.03^c^	1.65 ± 0.04^b^	1.7 ± 0.1^b^	2.0 ± 0.1^a^	2.01 ± 0.01^a^	2.0 ± 0.2^a^	2.1 ± 0.1^a^
Fat (g/100 g)	30.2 ± 1.3^a^	18.4 ± 0.4^b^	17.9 ± 0.2^bc^	17.0 ± 0.2^bcd^	16.3 ± 0.3^cd^	16.3 ± 0.4^d^	15.8 ± 0.3^d^
Ash (g/100 g)	2.9 ± 0.3^a^	2.8 ± 0.1^a^	2.9 ± 0.1^a^	2.8 ± 0.1^a^	2.81 ± 0.03^a^	2.6 ± 0.1^a^	2.6 ± 0.3^a^
Total fibre (g/100 g)	6.9 ± 0.2^c^	7.1 ± 0.2^c^	6.78 ± 0.04^c^	8.5 ± 0.3^ba^	8.2 ± 0.2^b^	8.8 ± 0.2^a^	8.9 ± 0.2^a^
Remaining carbohydrates (g/100 g)	5.0 ± 0.7^b^	11 ± 1^a^	11.7 ± 0.6^a^	11 ± 1^a^	11.1 ± 0.5^a^	11.1 ± 0.5^a^	11.5 ± 0.1^a^
Salt (g/100 g)	1.22 ± 0.03^c^	2.2 ± 0.1^a^	2.27 ± 0.03^a^	1.8 ± 0.1^b^	1.8 ± 0.1^b^	1.8 ± 0.1^b^	1.7 ± 0.1^b^
Vitamin E (mg/100 g)							
α-Tocopherol	10.3 ± 0.2^a^	8.8 ± 0.1^b^	9.6 ± 0.2^ba^	6.6 ± 0.6^c^	6.9 ± 0.2^c^	6.5 ± 0.5^c^	6.1 ± 0.2^c^
β-Tocopherol	0.371 ± 0.001^a^	0.37 ± 0.02^a^	0.36 ± 0.04^a^	0.30 ± 0.02^b^	0.26 ± 0.01^b^	0.30 ± 0.01^b^	0.29 ± 0.01^b^
*γ*-Tocopherol	0.64 ± 0.01^a^	0.60 ± 0.01^ba^	0.56 ± 0.02^b^	0.40 ± 0.03^c^	0.432 ± 0.001^c^	0.41 ± 0.03^c^	0.40 ± 0.02^c^
∑ Tocopherols	11.4 ± 0.3^a^	9.8 ± 0.1^b^	10.5 ± 0.2^ba^	7.3 ± 0.6^c^	7.6 ± 0.2^c^	7.2 ± 0.5^c^	6.8 ± 0.2^c^
TPC (g GAE/100 g)	0.20 ± 0.01^d^	0.43 ± 0.01^c^	0.45 ± 0.01^c^	0.56 ± 0.01^b^	0.56 ± 0.02^b^	0.64 ± 0.02^a^	0.64 ± 0.02^a^
Hydroxytyrosol (g/100 g)	n.d.	n.d.	n.d.	0.032 ± 0.001^ba^	0.03 ± 0.01^b^	0.038 ± 0.001^a^	0.04 ± 0.01^a^
FRAP (g FSE/100 g)	0.19 ± 0.01^d^	0.48 ± 0.02^c^	0.49 ± 0.02^c^	0.62 ± 0.02^b^	0.64 ± 0.02^b^	0.82 ± 0.03^a^	0.83 ± 0.03^a^
DPPH^•^-SA (g TE/100 g)	0.17 ± 0.01^d^	0.37 ± 0.02^c^	0.34 ± 0.04^c^	0.47 ± 0.03^b^	0.45 ± 0.03^b^	0.53 ± 0.03^a^	0.54 ± 0.02^ba^
Dry weight							
Protein (g/100 g)	2.42 ± 0.08^c^	4.0 ± 0.1^b^	4.1 ± 0.2^b^	5.0 ± 0.2^a^	4.97 ± 0.02^a^	5.0 ± 0.4^a^	5.1 ± 0.3^a^
Fat (g/100 g)	65.4 ± 2.9^a^	44.9 ± 0.9^b^	43.8 ± 0.5^bc^	41.4 ± 0.4^bcd^	40.4 ± 0.6^cd^	40 ± 1^d^	38.6 ± 0.6^d^
Ash (g/100 g)	6.3 ± 0.6^a^	6.8 ± 0.3^a^	7.1 ± 0.3^a^	6.8 ± 0.3^a^	6.9 ± 0.1^a^	6.3 ± 0.3^a^	6.4 ± 0.7^a^
Total fibre (g/100 g)	15.0 ± 0.5^d^	17.3 ± 0.4^c^	16.5 ± 0.1^c^	20.8 ± 0.7^ba^	20.2 ± 0.6^b^	21.6 ± 0.5^a^	21.8 ± 0.4^a^
Remaining carbohydrates (g/100 g)	11 ± 3^b^	27 ± 1^a^	28.5 ± 0.4^a^	26.1 ± 1.4^a^	27.5 ± 0.1^a^	27 ± 2^a^	28 ± 1^a^
Salt (g/100 g)	2.65 ± 0.06^c^	5.48 ± 0.17^a^	5.55 ± 0.08^a^	4.27 ± 0.20^b^	4.54 ± 0.14^b^	4.28 ± 0.23^b^	4.20 ± 0.22^b^
Vitamin E (mg/100 g)							
α-Tocopherol	22.4 ± 0.5^a^	21.8 ± 0.3^a^	23.4 ± 0.4^a^	16.2 ± 1.4^b^	16.8 ± 0.4^b^	15.9 ± 1.2^b^	15.0 ± 0.5^b^
β-Tocopherol	0.80 ± 0.01^ba^	0.90 ± 0.05^a^	0.89 ± 0.09^a^	0.74 ± 0.05^cb^	0.63 ± 0.02^c^	0.73 ± 0.02^cb^	0.70 ± 0.01^cb^
γ-Tocopherol	1.40 ± 0.02^a^	1.48 ± 0.03^a^	1.38 ± 0.04^a^	0.98 ± 0.05^b^	1.051 ± 0.001^b^	1.00 ± 0.07^b^	0.97 ± 0.06^b^
∑ Tocopherols	24.6 ± 0.6^a^	24.2 ± 0.2^a^	25.6 ± 0.3^a^	17.9 ± 1.6^b^	18.5 ± 0.4^b^	17.7 ± 1.2^b^	16.6 ± 0.5^b^
TPC (g GAE/100 g)	0.43 ± 0.02^d^	1.05 ± 0.01^c^	1.10 ± 0.01^c^	1.37 ± 0.02^b^	1.38 ± 0.05^b^	1.56 ± 0.04^a^	1.56 ± 0.04^a^
Hydroxytyrosol (g/100 g)	n.d.	n.d.	n.d.	0.08 ± 0.01^ba^	0.07 ± 0.01^b^	0.10 ± 0.01^a^	0.09 ± 0.01^a^
FRAP (g FSE/100 g)	0.40 ± 0.02^d^	1.16 ± 0.05^c^	1.19 ± 0.05^c^	1.51 ± 0.05^b^	1.59 ± 0.05^b^	2.00 ± 0.07^a^	2.04 ± 0.07^a^
DPPH^•^-SA (g TE/100 g)	0.38 ± 0.03^d^	0.91 ± 0.06^c^	0.85 ± 0.09^c^	1.15 ± 0.06^b^	1.12 ± 0.07^b^	1.29 ± 0.08^a^	1.33 ± 0.04^a^
Fatty acids (in relative %)							
Palmitic acid (C16:0)	12.60 ± 0.02^b^	13.29 ± 0.05^a^	13.14 ± 0.03^a^	13.07 ± 0.06^a^	13.0 ± 0.2^a^	13.3 ± 0.2^a^	13.1 ± 0.1^a^
Palmitoleic acid (C16:1)	1.00 ± 0.01^a^	0.92 ± 0.03^ba^	0.91 ± 0.02^ba^	0.90 ± 0.03^ba^	0.91 ± 0.06^ba^	0.93 ± 0.07^ba^	0.85 ± 0.06^b^
Stearic acid (C18:0)	2.88 ± 0.03^a^	2.46 ± 0.03^c^	2.47 ± 0.04^c^	2.59 ± 0.04^cb^	2.49 ± 0.08^c^	2.76 ± 0.07^ba^	2.6 ± 0.1^cb^
Oleic acid (C18:1*n*9*c*)	76.26 ± 0.06^a^	74.6 ± 0.3^b^	74.6 ± 0.1^b^	74.4 ± 0.1^b^	74.6 ± 0.2^b^	74.2 ± 0.2^b^	74.6 ± 0.3^b^
Linoleic acid (C18:2*n*6*c*)	5.82 ± 0.08^b^	7.3 ± 0.4^a^	7.42 ± 0.06^a^	7.60 ± 0.05^a^	7.62 ± 0.02^a^	7.4 ± 0.2^a^	7.5 ± 0.1^a^
α-Linolenic acid (C18:3*n*3)	0.72 ± 0.01^a^	0.81 ± 0.08^a^	0.81 ± 0.05^a^	0.83 ± 0.05^a^	0.69 ± 0.06^a^	0.73 ± 0.06^a^	0.78 ± 0.06^a^
∑SFA	15.99 ± 0.07^b^	16.24 ± 0.05^ba^	16.09 ± 0.03^ba^	16.1 ± 0.1^ba^	15.9 ± 0.3^b^	16.6 ± 0.3^a^	16.1 ± 0.2^ba^
∑MUFA	77.47 ± 0.05^a^	75.7 ± 0.3^b^	75.7 ± 0.1^b^	75.4 ± 0.1^b^	75.8 ± 0.2^b^	75.3 ± 0.1^b^	75.6 ± 0.3^b^
∑PUFA	6.54 ± 0.08^b^	8.1 ± 0.3^a^	8.23 ± 0.08^a^	8.4 ± 0.1^a^	8.31 ± 0.07^a^	8.1 ± 0.2^a^	8.2 ± 0.1^a^
MUFA/PUFA	11.9 ± 0.1^a^	9.4 ± 0.4^b^	9.2 ± 0.1^b^	9.0 ± 0.1^b^	9.1 ± 0.1^b^	9.3 ± 0.2^b^	9.2 ± 0.2^b^

The results are presented as mean ± standard deviation. Different superscript letters within each line represent significant differences between samples (*p* < 0.05). TPC, Total phenolic content; GAE, Gallic acid equivalents; FRAP, Ferric reducing antioxidant power; FSE, ferrous sulphate equivalents; DPPH^•^-SA, 2,2-Diphenyl-1-picrylhydrazyl radical scavenging activity; TE, Trolox equivalents; SFA, Saturated fatty acids; MUFA, Monounsaturated fatty acids; PUFA, Polyunsaturated fatty acids; n.d., not detected. Heptadecanoic (C17:0), arachidic (C20:0), cis-11-Eicosenoic (C20:1n9), behenic (C22:0) and lignoceric (C24:0) acids were less than 0.5%.

Among the lab-produced samples, moisture, ash, and carbohydrate contents were similar (*p* > 0.05). The incorporation of 25% PastOPP significantly reduced the total fat content from ≈18.2 g/100 g fw in the controls to ≈16.1 g/100 g fw in P25 and P25*. The fat of P20* and P25* is particularly high in MUFA (approximately 76%) and presents a MUFA/PUFA ratio of 9.1–9.2. Oleic acid was the predominant MUFA in the pâtés (74–76%), which is desirable, since it contributed to health benefits such as reducing inflammation, lowering LDL cholesterol levels, and protecting against mitochondrial dysfunction and insulin resistance (36, 37). Oleic acid was followed by palmitic (around 13%) and linoleic (7–8%) acids. These results reflect the fatty acid profile of the main ingredients used in the formulations: table olives (38), EVOO (39), and PastOPP (6).

The fatty acid composition of PastOPP observed in the present study is consistent with previously published data, which reports oleic acid as the most abundant (approximately 74%) fatty acid, followed by palmitic (around 11%) and linoleic acids (about 9%) (6). In general, P0, P20, and P25 had similar profiles (Table 2), while the commercial sample presented a significantly higher MUFA/PUFA ratio (12.0 vs. 9.0) and ∑MUFA (77% vs. 75–76%) alongside a slightly lower PUFA sum (7% vs. 8%). These differences can be attributed to the distinct ingredient compositions of the samples, particularly the higher proportion of olives and olive oil in the commercial pâté compared with the other samples. Despite this, the pâtés incorporating PastOPP still retained a high MUFA/PUFA ratio of 9.0–9.3, suggesting good oxidative stability and longer shelf lives (40).

Regarding total protein, PastOPP incorporation significantly increased its content in comparison to the controls (around 1.7 vs. 2.0–2.1 g/100 g), both with and without HPP treatment. However, no significant differences were observed between P20 and P25, and between P20* and P25* regarding this parameter (*p* > 0.05). Although these products have a low protein content (around 1.1–2.1 g/100 g fw), it would be relevant to evaluate the amino acid profile and essential amino acid scores in future analysis.

Since dietary fiber is not digested by the enzymes of the small intestine, a daily intake of 25–35 g is recommended for adults as part of a healthy, balanced diet due to its beneficial effects on cardiovascular health, body weight management, and digestive function (41). The incorporation of PastOPP led to a significant increase in total dietary fiber. According to the European Regulation, a food product is classified as “high in fiber” when it has at least 6 g fiber/100 g (42), so considering the registered contents (8.2–8.9 g/100 g), both P20* and P25* earn this claim. The high fiber content of these samples is a quality attribute, owing to the previously mentioned health-related properties of this macronutrient.

The incorporation of PastOPP significantly reduced the salt content (*p* < 0.05). In fact, higher salt levels were observed in P0 compared to P20 and P25, as well as P0* compared to P20* and P25* (*p* < 0.05). However, no significant differences between P20 and P25, nor between P20* and P25* were observed. The World Health Organization recommends limiting daily sodium intake to less than 2 g (≈5 g of salt) for adults (43). In Portugal, the average daily sodium intake is 2.962 g (≈7.4 g of salt), with men consuming more than women (3.431 vs. 2.547 g/day, respectively) (44), both exceeding the recommended values. Although these products are intended for occasional consumption in small portions (20 g), the high salt content in P20* and P25* can be reduced in future formulations, considering these recommendations. To address this issue, strategies such as the use of salicornia, a plant that can serve as a natural, low-sodium alternative to salt (45), or mineral salts (e.g., KCl, K_2_SO_4_, CaCl_2_, and MgCl_2_) could be considered (46).

Vitamin E prevents oxidative processes and free radical production. It protects polyunsaturated fatty acids (PUFA, 6.5–8.4%) from oxidation by halting the lipid peroxidation chain reaction. Since humans cannot synthesize vitamin E, it must be obtained through the diet. Its deficiency can lead to ataxia and enhance the risk of developing fat malabsorption-related conditions (47). Therefore, it should be consumed daily, with the recommended levels of *α*-tocopherol being 13 mg and 11 mg for adult males and females, respectively (48). All the pâtés incorporating PastOPP exhibited significantly lower levels of total vitamin E compared to CP, P0, and P0* (6.8–7.6 vs. 9.8–11.4 mg/100 g fw). This result can be attributed to the partial replacement of black oxidized olives with PastOPP in the initial formulations. As PastOPP is a by-product of olive oil extraction, and vitamin E is a liposoluble compound primarily found in olives, most of this vitamin is transferred to the EVOO fraction during oil production. Consequently, olive pomace, which contains only residual amounts of fat, is depleted in vitamin E, thereby explaining the reduced levels observed in the PastOPP-enriched samples. Despite this reduction, the pâtés incorporating PastOPP still represent a noteworthy source of vitamin E, mostly *α*-tocopherol (Table 2). Table olives contain α-tocopherol levels of up to 9 mg/100 g of edible portion (49), and as the main ingredient of the pátês, they constituted the primary source of this isomer. Moreover, the intensive processing involved in the production of black oxidized olives does not significantly affect this parameter, allowing high *α*-tocopherol levels to be retained (38). Tocotrienols, however, were not detected in the analysed samples, consistent with prior findings (38). In the developed pátês, the α-tocopherol contents were 6.9 and 6.2 mg/100 g fw, for of P20* and P25*, respectively. This means that a 20 g serving of P20* would provide 10.6 and 12.5% of the recommended daily intake for men and women, respectively. Similarly, 20 g of P25* would provide 9.5% of the recommended daily intake for men and 11.3% for women. Overall, all pâtés analyzed in this study can be considered suitable sources of α-tocopherol, despite the slight reduction observed in PastOPP-enriched samples.

Phenolic compounds are antioxidants that interact with the salivary glycoproteins, being responsible for the bitterness of fruits (50). Table olives are reported as rich sources of antioxidants (51). Consequently, ripe olives, if intended to be consumed on their own, must undergo fermentation or curing processes to reduce the content of bitter compounds before consumption (52). However, table olive processing can cause a loss of phenolic compounds, which can lead to a reduction of the antioxidant activity (53). In the present study, black oxidized olives were used to prepare the pátês. These olives are obtained through Californian-style processing, during which oleuropein, ligstroside, and verbascoside are hydrolyzed into non-bitter phenolic compounds during the lye treatment, resulting in lower TPC compared to other types of processed table olives (49, 52, 54). A previous study reported TPC ranging between 1.2 and 10.8 mg GAE/g pulp dw in commercial table olives darkened by oxidation (53).

EVOO, another ingredient used in the formulations, is a well-known source of phenolic compounds, with TPC values ranging between 12.7 and 17.7 mg GAE/100 g (39). These compounds contribute to EVOO’s health-promoting properties, including antimicrobial and antitumoral effects, which have also been reported for olive pomace (5, 8). Olive pomace presents high TPC levels as previously reported: 3.08 g GAE/100 g dw (6), 4.08 g GAE/100 g dw (7), and 3.92 g GAEs/100 g dw (9).

According to Table 2, the CP had a significantly lower TPC than all the lab-produced pâtés, a result strongly dependent on formulation differences. CP contains black olives (83%), olive oil, honey, and aromatic herbs, whereas the base formulation (P0) contains a lower proportion of black olives (75%) and includes vinegar (10%) and garlic powder (1%) instead of honey. PastOPP incorporation resulted in a significant increase of the TPC. As expected, due to the higher percentage of PastOPP incorporated, P25* had a significantly higher TPC than P20* (*p* < 0.05).

Numerous studies demonstrate that EVOO (39) and table olives (49) are the main sources of hydroxytyrosol (HT) in plant matrices. However, the Californian-style processing technique (used to produce the black oxidized olives employed in these pâtés) results in table olives with the lowest TPC, particularly with respect to HT content (54). Rocha et al. (49) reported that compared to other table olives, the black ripe olives have minimal or null hydroxytyrosol content. This is because HT levels in olives decrease markedly during the lye treatment steps, and the compound is further oxidized during the ferrous gluconate treatment used for color stabilization (54). According to Table 2, HT was only detected in the pâtés incorporating PastOPP (P20, P20*, P25, and P25*) which confirms that the olives were not a contributor of HT. Notably, EVOO also did not contribute with levels of this compound.

The European Food Safety Authority (EFSA) has authorized a health claim for olive oil polyphenols based on their beneficial effects on cardiovascular health, recommending a daily intake of 5 mg of hydroxytyrosol and its derivatives from olive oil to support a balanced diet (55). In fact, biscuits enriched with 5.25 mg of HT/30 g allowed a significant reduction of oxidized LDL plasma levels (56). P25* had a significantly higher hydroxytyrosol content than the P20* in both fw and dw (*p* < 0.05). Nevertheless, both can be considered olive pâtés rich in HT. Specifically, a 20 g serving of P20* provides approximately 6 mg of HT, while the same portion of P25* contains about 8 mg. These values indicate that consuming either pâté could contribute to the previously described health benefits associated with HT intake.

The antioxidants found (vitamin E, TPC, and HT) contribute to the antioxidant activity of the olive pâtés, assessed by FRAP and DPPH^●^-SA assays (Table 2). CP registered significantly lower antioxidant activities than the other samples, reflecting its lower TPC and the absence of HT. In contrast, PastOPP incorporation significantly improved the antioxidant activities of the pâtés compared to the controls. Among the PastOPP-enriched samples, P25* had a significantly higher antioxidant activity in both assays than P20*, corresponding to its higher TPC, resulting from the greater proportion of PastOPP.

In this study, HPP was applied to lab-formulated olive pâtés containing PastOPP to investigate its effects on chemical composition, bioactive compound retention, antioxidant activity, and sensory attributes. In general, no significant differences were found between HPP-processed and unprocessed pâtés in terms of macronutrients, fatty acids, vitamin E, TPC, hydroxytyrosol content, and antioxidant activity (Table 2), indicating that HPP processing did not significantly impact the nutritional composition of olive pâtés, which is in accordance with several studies with other food matrices (57–64) and confirms the advantages of using this non-thermal food preservation technology to inactivate microorganisms, helping to preserve the food product and extend shelf life (24). In the future, studies on stability and shelf life should be conducted to determine the safe consumption period of these olive pâtés.

#### 3.1.1 Sensory evaluation of olive pâtés

Participants covered a wide age range, with the majority being between 18 and 57 years. The age distribution across subgroups as follows: 18–25 years (27%), 26–33 years (15%), 34–41 years (13%), 42–49 years (13%), and 50–57 years (14%). Lower representation was observed among younger participants (<18 years, 9%) and older age groups, including 58–65 years (5%), 66–73 years (2%), and ≥74 years (2%). Regarding gender, females constituted most participants (55%), while males represented 45%. In terms of occupation, most participants were workers (54%) or students (33%), with smaller proportions of retired individuals (6%), unemployed individuals (5%), student workers (1%), and domestic workers (1%).

#### 3.1.2 Check-all-that-apply (CATA) test

The CATA list included 34 terms categorized by sensory attributes: appearance, texture, odor, mouthfeel, flavor, and taste. For each pâté, the frequency of selection for each term by consumers was calculated by dividing the number of assessors who selected that term by the total number of assessors (Table 3). Within each sensory category, the least and most frequently selected attributes were as follows: “not shiny” and “shiny” (appearance); “spicy” and “olive” (odor); “liquid” and “grainy” (spreadability texture); “salty” and “olive” (taste); and “liquid” and “creamy” (mouthfeel).

**Table 3:** Frequency of terms of the CATA test selected by consumers to describe the olive pâtés (CP, P0*, P20*, P25*) and their ideal olive pâtés (IP).

Attributes		Olive pâtés				
		CP	P0*	P20*	P25*	IP
Aspect	“Not shiny”	9^ab^	7^ab^	8^ab^	13^b^	0^a^
	“Shiny”	50^a^	65^ab^	75^b^	61^ab^	70^b^
	“Light”	40^b^	9^a^	4^a^	3^a^	12^a^
	“Dark”	29^a^	60^b^	60^b^	70^b^	57^b^
	“Grainy”	14^a^	44^c^	31^bc^	39^bc^	26^ab^
	“Oily”	49^b^	22^a^	26^a^	23^a^	12^a^
Odor	“Aromatic”	8^a^	31^bc^	26^b^	23^b^	43^c^
	“Vinegary”	11^a^	36^bc^	49^c^	37^bc^	21^ab^
	“Olive oil”	18^a^	47^b^	26^a^	34^ab^	45^b^
	“Olive”	33^a^	63^bc^	62^bc^	58^b^	77^c^
	“Spicy”	0^a^	4^ab^	3^ab^	6^ab^	9^b^
	“Rancid”	69^b^	1^a^	1^a^	0^a^	0^a^
Texture (spread on a cracker)	“Spreadable”	48^b^	30^a^	35^ab^	31^a^	47^b^
	“Creamy”	45^ab^	31^a^	31^a^	31^a^	61^b^
	“Grainy”	32^a^	63^b^	53^b^	59^b^	27^a^
	“Liquid”	4^a^	2^a^	0^a^	0^a^	1^a^
	“Soft”	12^b^	4^ab^	8^ab^	0^a^	13^b^
	“Sticky”	16^b^	6^a^	9^ab^	10^ab^	2^a^
Taste	“Acidic”	2^a^	10^ab^	20^b^	22^b^	14^ab^
	“Astringent”	10^ab^	1^a^	12^ab^	18^b^	3^a^
	“Bitter”	8^a^	11^a^	48^b^	46^b^	8^a^
	“Vinegary”	5^a^	46^c^	34^bc^	22^ab^	33^bc^
	“Olive oil”	24^a^	54^bc^	42^b^	39^ab^	61^c^
	“Olive”	35^a^	79^bc^	79^bc^	63^b^	88^c^
	“Spices”	11^a^	65^c^	50^bc^	36^b^	67^c^
	“Very bitter”	5^a^	3^a^	4^a^	15^b^	0^a^
	“Spicy”	0^a^	4^a^	4^a^	2^a^	18^b^
	“Rancid”	72^b^	0^a^	0^a^	1^a^	0^a^
	“Salty”	4^a^	1^a^	0^a^	0^a^	17^b^
Texture (mouth)	“Consistent”	9^a^	14^ab^	18^ab^	19^ab^	22^b^
	“Creamy”	58^bc^	40^a^	45^ab^	41^ab^	75^c^
	“Grainy”	29^ab^	53^c^	44^bc^	55^c^	21^a^
	“Liquid”	8^c^	7^bc^	1^ab^	0^a^	0^a^
	“Sticky”	16^b^	4^a^	4^a^	3^a^	2^a^

Results are presented in frequency of mention (%). Within rows, different lowercase superscript letters represent significant differences between the frequency of mention of attributes for each olive pâté (*p* < 0.05). CP, Commercial pâté; P0*, pâté processed by HPP; P20*, pâté with 20% PastOPP processed by HPP; P25*, pâté with 25% PastOPP processed by HPP; IP, Ideal pâté.

To determine whether consumers perceived significant differences among the pâtés, a Cochran’s Q test was conducted, followed by a post-hoc analysis using multiple pairwise comparisons through the critical difference (Sheskin) procedure, as applied in previous studies (33, 65). The results revealed that the term “liquid” was the only attribute not used to discriminate the pâtés (*p* > 0.05). In contrast, the remaining 33 attributes significantly differentiated the samples (*p* < 0.05). The high number of attributes used by consumers suggests that they detected differences in the sensory features of the different samples. However, not all attributes were deemed essential for distinguishing the samples.

According to Table 3, consumers selected the attributes “spicy” and “salty” (taste) with significantly higher frequency for the ideal olive pâté compared to the other samples (*p* < 0.05). This suggests that consumers perceive an ideal olive pâté to be spicier and saltier than the ones produced in this study.

Regarding texture, consumers prefer creamier and less grainy pâtés (Table 3). For the lab-produced pâtés, the preparation involved triturating the olives for 10 min, followed by mixing with PastOPP, vinegar, and EVOO for an additional 10 min, and a final 10-min mixing step after adding herbs, garlic powder, and salt. In future formulations, grinding time will be increased to 15 min at each step to achieve a creamier texture. By optimizing these grinding and mixing steps, a smoother texture more aligned with consumer preferences is expected. Additionally, both mechanical and sensory texture analyses will be incorporated in future work to more rigorously evaluate texture prior to final tasting.

**Figure 1:**
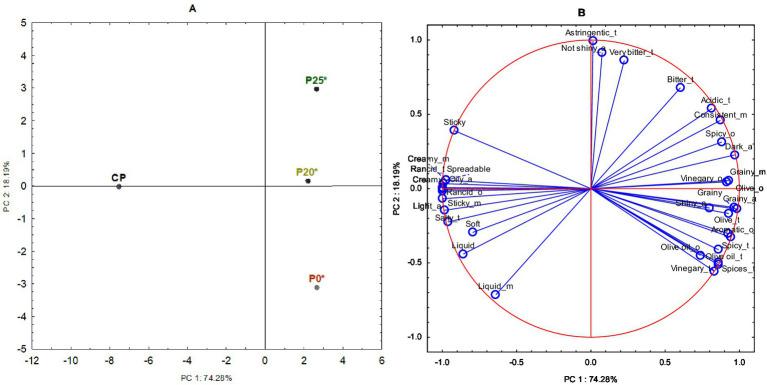
Principal component analysis (PCA) performed on the matrix of the absolute frequency of mention of each attribute for each tasted olive pâté. **(A)** Projection of the olive pâtés on the factor-plane; **(B)** Projection of the variables on the factor-plane. CP, commercial pâté; P0*, pâté processed by HPP; P20*, pâté with 20% PastOPP processed by HPP; P25*, pâté with 25% PastOPP processed by HPP; a, aspect; o, odor; t, taste; m, texture (mouth).

CATA results were analyzed using PCA to reduce data dimensionality (Figure 1). In this study, CATA data were first organized into a matrix containing the absolute frequency of each checked attribute for each olive pâté.

The PCA plot explained 92.47% of the dataset variability through the first two principal components (PC1 and PC2). PC1 accounted for over 74% of the total variability, while PC2 explained more than 18%. Examining PC1 (horizontal axis), it is evident that CP was projected on the opposite side, separated from P0*, P20*, and P25*. Additionally, PC2 (vertical axis) highlights differences among P0*, P20*, and P25*. Specifically, P20* and P25* are positioned on the positive side of both PC1 and PC2, while P0* is located on the bottom-right side. In contrast, CP is situated on the negative side of PC1 and on the origin in PC2. The position of CP and its associated attributes in the PCA indicates that consumers perceived this pâté as having a higher intensity of light and oily appearance, rancid odor, spreadable and creamy texture when spread on a cracker, rancid and salty flavors, and a creamy and sticky mouthfeel. Regarding lab-produced olive pâtés, P0* was associated with shiny and grainy appearance; aromatic, olive oil, and olive odors; grainy texture when spread on a cracker; and vinegary, olive oil, olive, spices, and spicy flavors. Conversely, P20* was more closely associated with a dark appearance, vinegary odor, bitter taste, and consistent and grainy mouthfeel texture. Meanwhile, P25* was mainly associated with a dull appearance and astringent, bitter, and very bitter tastes.

#### 3.1.3 Acceptability test

The development of new food products on an industrial scale heavily depends on sensory analysis, with the food industry commonly using consumer affective ratings to guide product development, improvement, and quality control processes. Product acceptance is influenced by genetics, age, gender, cultural practices, and education. It depends on the extent to which consumer needs and expectations are met, and satisfaction is achieved (13, 66). Although attitudinal measures of food acceptance may not always accurately predict actual consumption behavior, they are widely used to forecast consumer responses to innovative products because of their simplicity, speed, and ease of implementation. These tests typically measure the degree of liking based on sensory attributes, most often using a 9-point hedonic scale (13, 66). In the present study, this scale was employed to capture subtle differences in consumer liking, allowing a fine-grained assessment of sensory preferences.

**Figure 2:**
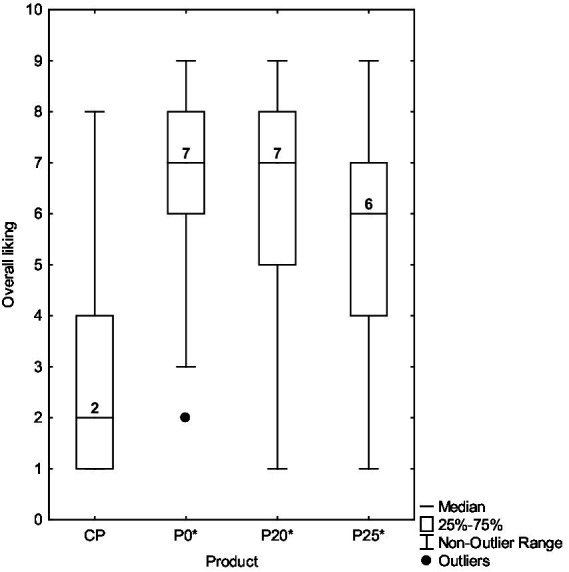
Boxplots for the acceptability of the pâtés: evaluation on a 9-point hedonic scale from 1 (“Dislike extremely”) to 9 (“Like extremely”). CP, commercial olive pâté; P0*, control pâté processed by HPP; P20*, pâté with 20% PastOPP processed by HPP; P25*, pâté with 25% PastOPP processed by HPP.

The acceptability test highlighted clear differences in consumer preferences among the four olive pâtés. CP was the least liked, with only 18% of consumers expressing a positive opinion and a significant proportion reporting dislike. In contrast, P0* was the preferred sample, with 86% of participants indicating liking, followed by P20*, which was liked by 74% of consumers. P25* also received positive feedback from 62% of participants but exhibited slightly more polarized opinions compared to P0* and P20*. Overall, P0* and P20* showed the highest levels of consumer acceptance, suggesting strong potential for commercial application. Consumer acceptability of the olive pâtés is illustrated using boxplots (Figure 2). As shown, P0* and P20* were the most appreciated samples (median = 7), followed by P25* (median = 6), whereas CP was the least appreciated (median = 2). Notably, the acceptability scores for the pâtés containing PastOPP showed substantial variability (ranging between 1 and 9), indicating that some assessors expressed strong liking and others clear dislike (67).

The Kruskal-Wallis test confirmed that acceptability ratings differed significantly among the olive pâtés (H (3) = 128.966, *p* < 0.05). Post-hoc pairwise comparisons revealed no significant differences between P0* and P20* or between P20* and P25* (*p* > 0.05). In contrast, significant differences were observed between P0* and P25*, as well as between CP and all the other pâtés (*p* < 0.05).

**Figure 3:**
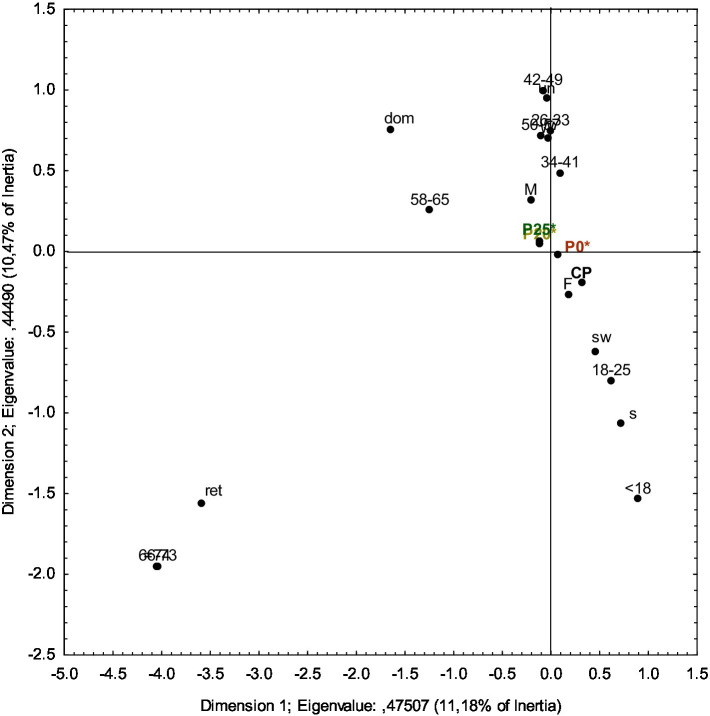
Correspondence analysis of the tasters’ scores on the acceptability of the olive pâtés, categorized by sex, age group, and occupation. CP, Commercial olive pâté; P0*, control pâté processed by HPP; P20*, pâté with 20% PastOPP processed by HPP; P25*, pâté with 25% PastOPP processed by HPP; F, female; M, male; dom, domestic; w, worker; s, student; sw, student worker; ret., retired.

Correspondence analysis (CA) was conducted to explore the relationship between consumer acceptability and the demographic characteristics of the assessors (sex, age group, and occupation) who evaluated the olive pâtés (Figure 3).

In Figure 3, the first and second dimensions clearly separate female consumers, projected in the bottom-right quadrant, from male consumers, who are projected in the top-left quadrant. With respect to occupation, students and student workers are projected in the bottom-right quadrant, whereas workers, unemployed, and domestic consumers are in the top-left quadrant. Retired consumers are in the bottom-left quadrant. Regarding age groups, consumers aged up to 25 years are projected in the bottom-right quadrant; those aged between 26–33 and 42–65 years are positioned in the top-left quadrant; those aged between 34 and 41 years are positioned in the top-right quadrant; and consumers older than 66 years are in the bottom-left quadrant.

With respect to the olive pâtés, CP and P0* are projected in the bottom-right quadrant, while P20* and P25* are in the top-left quadrant. On the right side of Dimension 1, CP and P0* are positioned closer to female consumers, suggesting that, overall, women assigned higher acceptability scores to these olive pâtés. In terms of age, both samples are closer to consumers aged 18–25 years, indicating that this age group generally rated these pâtés more favorably. Additionally, along the second dimension, these samples are positioned closer to student workers, suggesting higher acceptance among consumers with this occupation. Conversely, on the left side of Dimension 1, P20* and P25* are positioned closer to male consumers, indicating that, overall, men assigned higher acceptability scores to these olive pâtés. Regarding age, both samples are closer to consumers aged 50–57 and 58–65, suggesting greater acceptance among these age groups. Furthermore, their proximity to workers indicates that this occupational group generally rated these olive pâtés more favorably.

Considering that no significant differences in acceptability were found between P20* and P25*, it is relevant to evaluate whether consumers of a given gender, age, or occupation showed a preference for one sample over the other. According to the CA results (Figure 3), P20* and P25* are projected very close to each other, indicating no meaningful differences in acceptability between them. This observation is consistent with the hedonic acceptability scores, suggesting that both pâtés have promising commercial potential.

Phenolic compounds can influence the organoleptic characteristics of food products, as they contribute, for example, to bitterness and astringency (68). Many consumers perceive bitterness as an unpleasant sensory attribute, which may limit product consumption. For instance, the bitterness and other organoleptic properties of vegetables are often regarded as undesirable, posing challenges for dietary guidelines aimed at increasing vegetable intake (69). Nevertheless, phenolic compounds are frequently incorporated into food products to extend shelf life and enhance nutritional value, thereby improving overall product quality (68). Therefore, it was expected that the acceptability of the olive pâtés incorporating PastOPP (P20* and P25*) would be influenced by their higher TPC and hydroxytyrosol content compared with P0*.

On a 9-point hedonic scale, an acceptability score of 6 (the first point in the “like” category) is considered the minimum threshold for commercial or quality acceptance of a product (70). According to Figure 2, the median acceptability scores of P0*, P20*, and P25* reached acceptable levels (7, 7, and 6 points, respectively), whereas CP was substantially below this threshold (2 points). Therefore, the astringency caused by the phenolic content of PastOPP did not impair consumer acceptability, although a higher incorporation of PastOPP was associated with a lower median score. These findings further support the conclusion that both P20* and P25* can be considered olive pâtés with potential for commercial application, with P20* being the preferred formulation.

#### 3.1.4 Purchase intention

Many factors influence a consumer’s intention to purchase a food product beyond sensory appeal alone. Both initial purchase and repurchase decisions are shaped by variables such as price, brand positioning, market concept, packaging information, advertising strategies, consumer awareness, and perceived nutritional benefits. Consequently, assessing purchase intention through a blind sensory test presents inherent limitations, as key extrinsic factors, such as product price, competitive positioning, label claims, and promotional strategies, are not considered in this type of evaluation (15). Nevertheless, purchase intention is commonly assessed in studies involving newly developed food products (9, 10, 18). In the present study, a 5-point scale was used, as decisions regarding product purchase are typically less nuanced, and a shorter scale facilitates clear and straightforward responses compared to acceptability assessment, for which a 9-point scale was used.

The purchase intention of the olive pâtés (CP, P0*, P20*, and P25*) was evaluated by 100 consumers using a 5-point hedonic scale ranging from 1 (“Definitely would not buy”) to 5 (“Definitely would buy”). The results revealed significant differences among the samples. CP showed the lowest purchase appeal, with 59% of consumers indicating that they would “definitely not buy” the product and only 2% expressing strong purchase intent. In contrast, P0* achieved the highest purchase intention, with 31% of participants reporting that they would “definitely buy” it and 35% indicating that they would “probably buy” it.

**Figure 4:**
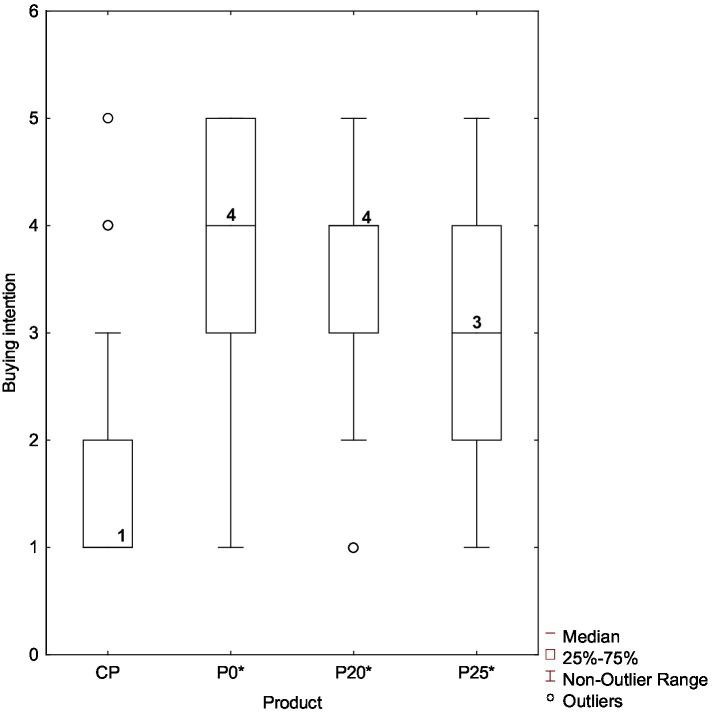
Boxplots for the purchase intention of olive pâtés: Evaluation on a 5-point hedonic scale from 1 (“Definitely would not buy”) to 5 (“Definitely would buy”). CP, Commercial olive pâté; P0*, Control pâté processed by HPP; P20*, Pâté with 20% PastOPP processed by HPP; P25*, Pâté with 25% PastOPP processed by HPP.

The pâtés incorporating PastOPP also demonstrated promising purchase potential. Both P20* and P25* received “definitely would buy” responses from 23% of consumers. However, P20* showed a slightly higher overall purchase intention. While P25* elicited more polarized responses, with 47% of consumers indicating a willingness to purchase, P0* and P20* emerged as the most promising samples, with 66 and 61% of consumers, respectively, expressing purchase intent. As shown in Figure 4, consumers exhibited greater interest in purchasing P0* and P20* (median = 4), followed by P25* (median = 3), whereas CP showed very low purchase interest (median = 1).

The Kruskal-Wallis test confirmed that purchase intention ratings differed significantly among the olive pâtés (H (3) = 116.720, *p* < 0.05). Post-hoc pairwise comparisons indicated no significant differences between P0* and P20* or between P20* and P25* (*p* > 0.05). In contrast, significant differences were observed between P25* and P0*, as well as between CP and all other pâtés (*p* < 0.05).

**Figure 5:**
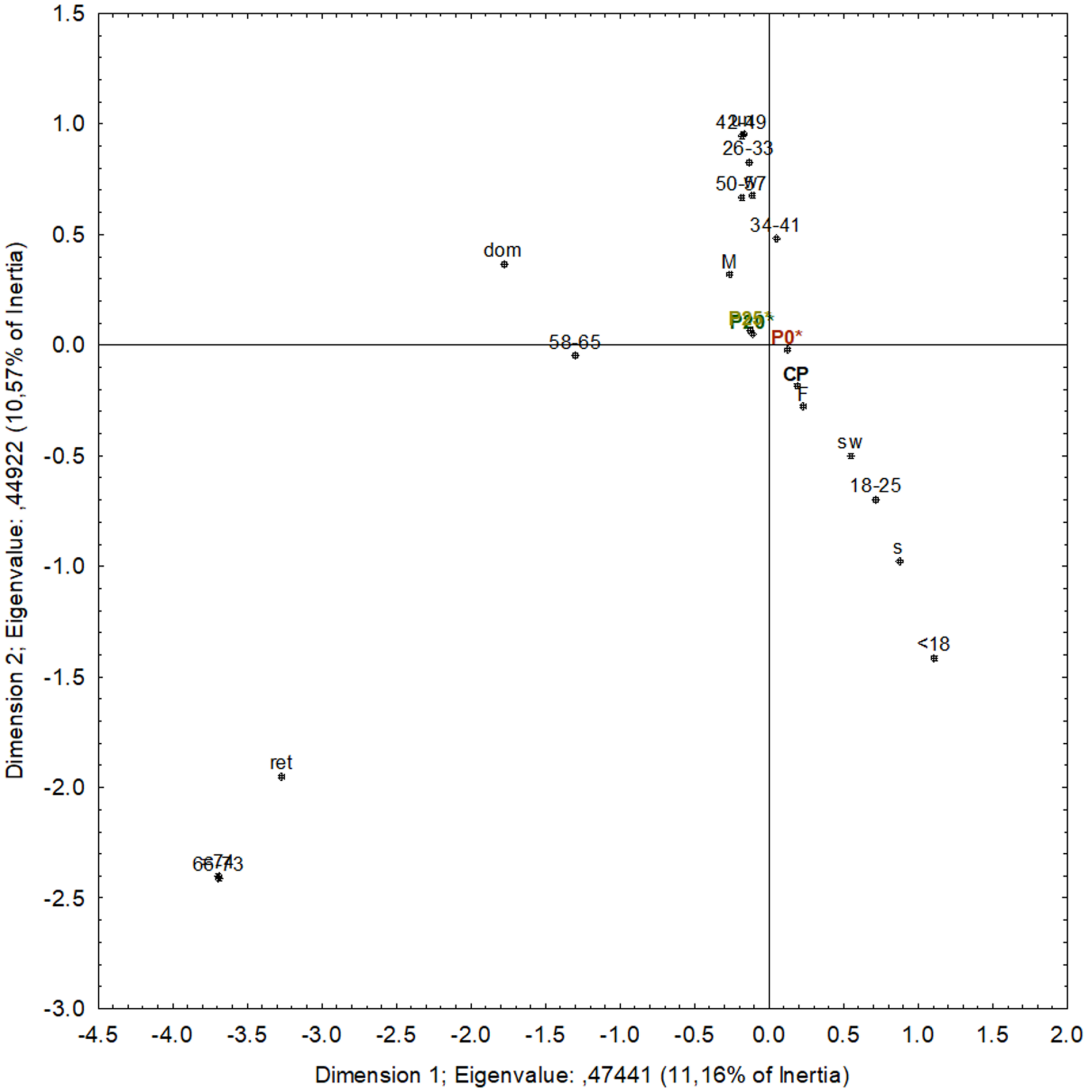
Correspondence analysis of the tasters’ scores on the purchase intention of the tasted olive pâtés, categorized by sex, age group, and occupation. CP, commercial olive pâté; P0*, control pâté processed by HPP; P20*, pâté with 20% PastOPP processed by HPP; P25*, pâté with 25% PastOPP processed by HPP; F, female; M, male; dom, domestic; w, worker; s, student; sw, student worker; ret., retired.

Correspondence analysis was also performed to explore the relationship between the purchase intention and the characteristics of the individuals (sex, age group, and occupation) who tasted the pâtés. As shown in Figure 5, female consumers are clearly separated from male consumers (bottom-right and top-left quadrants, respectively).

With respect to occupation, students and student workers are projected in the bottom-right quadrant, whereas workers, unemployed, and domestic consumers are in the top-left quadrant. Retired consumers are positioned in the bottom-left quadrant.

Regarding age, consumers younger than 25 years are projected in the bottom-right quadrant; those aged 26–33 and 42–65 years are in the top-left quadrant; consumers aged 34–41 years are positioned in the top-right quadrant; and consumers older than 66 years are projected in the bottom-left quadrant.

Concerning the olive pâtés, CP and P0* are projected in the bottom-right quadrant, while P20* and P25* are positioned in the top-left quadrant. On the right side of Dimension 1, CP and P0* are positioned closer to female consumers, suggesting that, overall, women reported higher purchase intention scores for these pâtés. In terms of age, both samples are closer to consumers aged 18–25 years, indicating that this age group generally showed greater purchase intention for these products. Additionally, along the second dimension, these samples are positioned closer to student workers, suggesting higher purchase intention among consumers with this occupation. Conversely, on the left side of Dimension 1, P20* and P25* are positioned closer to male consumers, indicating that, overall, men reported higher purchase intention scores for these pâtés. Regarding age, both samples are associated with consumers aged 50–57 and 58–65 years, suggesting greater purchase intention among these age groups. Furthermore, their proximity to workers indicates that this occupational group generally expressed higher purchase intention for these products.

Considering that no significant differences in purchase intention were detected between P20* and P25*, it is relevant to assess whether specific demographic groups showed a preference for one sample over the other. As observed in the correspondence analysis applied to acceptability data, Figure 5 shows that P20* and P25* are projected very close to each other, indicating that no meaningful differences in purchase intention can be identified between them across gender, age, or occupation. Therefore, both P20* and P25* can be considered olive pâtés with promising commercial potential.

## 4 Conclusion

PastOPP is a promising food ingredient owing to its low fat, high fiber, and hydroxytyrosol contents. Nevertheless, its bitter taste poses a challenge to its incorporation in food products. Laboratory-produced formulations containing different percentages of PastOPP (0, 20, and 25%: P0, P20, and P25, respectively) were compared to a commercial olive pâté (CP). Sensory evaluation involved HPP-treated pâtés to improve shelf life. HPP did not significantly alter the nutritional composition of the developed products in terms of macronutrients, vitamin E, fatty acids, TPC, hydroxytyrosol content, and antioxidant activity, thereby preserving their nutritional profile. Consequently, HPP did not significantly reduce bitterness, indicating that processing alone was insufficient to mitigate this sensory attribute. However, bitterness perception was effectively improved through the addition of masking ingredients such as herbs and spices.

The commercial pátê contained significantly lower moisture, protein, carbohydrate, salt, PUFA, TPC, and antioxidant activity compared to the other pâtés. Conversely, it exhibited higher fat content, ∑MUFA, and a higher MUFA/PUFA ratio. Notably, hydroxytyrosol was absent in CP. The HPP-processed pâtés with 20 and 25% PastOPP (P20* and P25*, respectively) could offer numerous health benefits due to high fiber content, significant vitamin E levels, and a favorable fatty acid profile. Both formulations also contained substantial levels of hydroxytyrosol, with a 20 g serving sufficient to meet the EFSA health claim.

Sensory analysis confirmed that P20* and P25* were well received by consumers. The CATA test indicated that P20* was associated with attributes such as a dark appearance, vinegary odor, bitter taste, and consistent texture, whereas P25* was linked to a less shiny appearance and more intense bitter and astringent flavors. Both products achieved commercial acceptability on a 9-point hedonic scale and received significantly higher acceptability scores than CP. Consumer acceptability and purchase intention scores for P20* and P25* did not differ significantly according to age, gender, or occupation. Although both formulations demonstrated promising commercial potential, P20* emerged as the preferred olive pâté. Both products align with current food trends focusing on health, sustainability, and convenience. These new products offer versatility as a snack or condiment for dishes like pasta and salads, making them a valuable addition to the food industry and contributing to the circular economy of the Portuguese olive oil industry. Future studies could include digestive analyses to better evaluate the bioavailability and functional properties of these newly developed formulations, particularly following HPP application.

## Data Availability

The original contributions presented in the study are included in the article/supplementary material, further inquiries can be directed to the corresponding authors.
